# ‘Beyond Cancer’ Rehabilitation Program to Support Breast Cancer Survivors to Return to Health, Wellness and Work: Feasibility Study Outcomes

**DOI:** 10.3390/curroncol30020174

**Published:** 2023-02-13

**Authors:** Dianne M. Sheppard, Moira O’Connor, Michael Jefford, Georgina Lamb, Dorothy Frost, Niki Ellis, Georgia K. B. Halkett

**Affiliations:** 1 Monash University Accident Research Centre, Monash University, Building 70, 21 Alliance Lane, Clayton 3800, Australia; 2 School of Population Health, Curtin University, Kent St, Bentley 6102, Australia; 3 Department of Health Services Research, Peter MacCallum Cancer Centre, 305 Grattan St, Melbourne 3000, Australia; 4 Australian Cancer Survivorship Centre, Peter MacCallum Cancer Centre, 305 Grattan St, Melbourne 3000, Australia; 5 Sir Peter MacCallum Department of Oncology, The University of Melbourne, Parkville 3010, Australia; 6 IPAR Rehabilitation, 485 La Trobe St, Melbourne 3000, Australia; 7 Healthy Working Lives Research Group, School of Public Health and Preventive Medicine, Monash University, Level 2, 533 St Kilda Road, Melbourne 3004, Australia; 8 Curtin School of Nursing/Curtin Health Innovation Research Institute, Curtin University, Kent St, Bentley 6102, Australia

**Keywords:** breast cancer survivorship, sustainable work, return to work, vocational rehabilitation, multimodal, feasibility study

## Abstract

More women are returning to work following breast cancer treatment. Our team designed ‘Beyond Cancer’, a multimodal rehabilitation program to support breast cancer survivors to return to work. This study aimed to determine the feasibility of the intervention from the breast cancer survivor, employer and occupational rehabilitation provider perspectives. The feasibility design focused on implementation, acceptability and preliminary indications of efficacy. Primary outcome measures included work status, work capacity and perceived support at work. Responses were compared with an historical usual care group of mixed cancer survivors. The tailored intervention was delivered over 33 weeks (on average) by trained occupational rehabilitation consultants. Eighty-four women with breast cancer (mean age = 50.8 years, SD = 8.24) who were unable to work in their pre-diagnosis capacity for >3 months participated. Results provided preliminary indications of efficacy for primary work outcomes, including work capacity relative to the historical usual care group, and some secondary biopsychosocial variables (physical fatigue, return to work expectations). The intervention was acceptable, demonstrated strong participant engagement and high satisfaction. Feasibility has been demonstrated for this multimodal intervention focused on returning to sustainable work for women with breast cancer. Future research is required with people diagnosed with other cancer types to demonstrate broader implementation.

## 1. Introduction

Breast cancer is the most common cancer in developed nations, with over two million cases diagnosed per year globally [1]. In Australia, approximately 20,000 women are diagnosed with breast cancer annually [2]. Currently, Australia has a 5-year relative survival rate for breast cancer of 92% [2,3]. England and Wales combined reported an 85% 5-year relative survival rate for 2013–2017, similar to that of Denmark, Austria and Germany [4]. Relative survival rates for all cancers combined have been improving over the last 30 years in Australia, with 70% of all patients diagnosed with cancer (excluding squamous and basal cell carcinomas) surviving at least 5 years after diagnosis, improving from 51% around 30 years ago [2,3]. Such gains are likely due to better diagnostic methods, earlier detection and improvements in treatment [5].

Across developed nations, approximately 40–55% of cancer cases occur in people of working age (18–65 years) [6,7]. In the U.S., individuals are most frequently diagnosed with breast cancer between the ages of 45 and 64 years [7]. In addition, support to remain at work or return to sustainable work is a recognised gap in the continuum of survivorship care [8,9]. There is also some early evidence from a recent Taiwanese study that RTW may be protective in the context of cancer-related mortality, with returning to work found to be associated with improved survival from breast cancer [10]. These studies and statistics clearly justify prioritising research that addresses issues concerning sustainable employment for cancer survivors, and in particular, breast cancer survivors [3,11].

### 1.1. Barriers to Returning to Sustainable Work for Women with Breast Cancer

For cancer survivors, common persistent experiences that affect work ability and readiness to return to work include fatigue, pain, cognitive impairments and fluctuations, anxiety and depression, emotional adjustment issues, fear of recurrence, body image perception and medication/radiation side effects [12,13,14,15]. A recent qualitative study by Urquhart et al. interviewed survivors (*n* = 13 participants with different diagnoses) at three time points after treatment and identified the following key themes: the need for support to enable RTW; others’ limited understanding of the long-term impacts of having a cancer diagnosis and receiving treatment; worries and self-doubts about returning to work; and how survivors’ perspectives on life and work changed [16].

Due to these barriers and challenges experienced by survivors, we see increased work absence, longer time to transition back to pre-diagnosis hours and tasks, higher likelihood of job change, lower income, and higher rates of unemployment and early retirement for those looking to sustain or return to work (RTW) following cancer compared to the general population [17,18,19], even for the younger working age group [20]. Further, women report unmet needs in relation to returning to work following treatment for breast cancer [21]. Building work readiness and transitioning back to meaningful and sustainable work for women with breast cancer can be especially complex, for example when facing competing priorities such as family responsibilities and potential role changes at home and work [20,22]. A more supportive and flexible workplace is likely to reduce the impact of some of these challenges [23,24] by facilitating the reintegration process for the employee [25]. There is a positive association between higher levels of workplace support and rates of cancer survivors returning to work [11], and problems at work (poor treatment, discrimination, poor accommodations) are associated with a reduced work ability, and also with losing or leaving a job because of cancer [26].

### 1.2. The Need for RTW Support Interventions

For the individual, sustaining work or returning to work assists with restoring social networks and a sense of being valued, regaining a sense of normality and re-establishing routines [9,23,27]. Recent qualitative findings reinforced that there is a critical need for supportive resources designed for cancer survivors generally, as well as their employers and/or health care practitioners in order to optimise sustainable work situations [28]. Many breast cancer survivors express the desire to remain at work or RTW during or following treatment [9], with a recent workforce participation study clearly demonstrating a specific need for support to return to work for Australian women with breast cancer [27]. A recent study found positive psychological outcomes associated with participating in a nurse-led, group-based psychoeducational intervention for young breast cancer patients [29]; however, recently published work has also emphasised that the development of individualised and RTW-focused interventions for women with breast cancer is a priority [30,31]. Employers and society are also likely to benefit from developing effective occupational rehabilitation interventions that facilitate the return to sustainable, appropriate work.

The 2017 National Cancer Policy Forum Workshop highlighted that many opportunities remain to improve quality of life and outcomes for cancer survivors, particularly initiatives that facilitate returning to life, work and school that take a whole-person approach by addressing the psychosocial needs of the survivor as well as the physical [32]. Treating clinicians who provide follow-up care post treatment may not have the capacity to extend their services to provide the required support and guidance to foster the transition to work readiness [8]. In contrast, vocational rehabilitation consultants working in the occupational rehabilitation (OR) setting are well placed to provide this support. In the life insurance sector, cancer is the third most common cause of life insurance claims in Australia [33], behind musculoskeletal and mental health issues; thus, facilitating recovery, rehabilitation and a return to wellness and work for these populations is a priority for employers and society.

The ‘cancer and work’ model [34] provides a useful framework for the conceptualisation of the broad range of individual and workplace factors influencing work outcomes for cancer survivors. Aligned with this systems framework [34], as well as the more recent occupational therapy model for RTW for those with breast cancer [35], Beyond Cancer was developed using the MRC framework for complex interventions [36], as a tailored occupational rehabilitation intervention that enables the identification and modification of the impact of psychosocial and workplace factors that could hinder transitioning to sustainable work and wellness for breast cancer survivors.

Feasibility study aims and objectives.

The overall objective of this study was to determine the feasibility of the Beyond Cancer program, from the breast cancer survivor, employer and OR provider perspectives, to support the transition back to sustainable work and living well for breast cancer survivors. Secondary objectives focused on determining the acceptability of the intervention and preliminary indications of efficacy. (Note that the insurance perspective was also important for this feasibility study and was obtained instead through their participation in an earlier pilot, the steering committee of this feasibility stage, and advice during the co-development of the intervention. Their strong support from early stages was integral to the study’s success, particularly with engaging the state-based insurers).

## 2. Materials and Methods

A feasibility study was conducted. In this methodology section we describe the sample, intervention development and how the data were analysed.

### 2.1. Proposed Sample

Proposed participants for the feasibility study were 120 breast cancer survivors aged 18–65 years unable to work in their regular (pre-diagnosis) capacity for at least 3 months due to breast cancer and its treatments (note that the actual participating sample is described below under results, as part of the feasibility evaluation). Breast cancer survivor participants were to be recruited over a period of 10 months nationally through the life insurance sector, the sickness absence claims database of recruited public and private national employers and direct referrals from cancer specialist hospitals such as Peter MacCallum in Melbourne.

The trained OR consultants confirmed that all individuals referred met the following eligibility criteria:

1. Working prior to diagnosis and treatment, but not yet RTW at full capacity;
2. Ready to participate in the intervention in the context of their general health and other circumstances in order to build their ‘work-readiness’ (note that this was difficult to determine as perceptions of readiness varied greatly across individuals and were not clearly associated with a particular stage of rehabilitation).

CONSORT guidelines were followed for recruitment and monitoring of response rates and withdrawals [37].

### 2.2. Review of Patient Case Notes

As an additional measure of representativeness of the breast cancer cohort referred to Beyond Cancer, OR case notes were examined to obtain a high-level understanding of the primary challenges to social participation, including work, for the breast cancer survivor program participants (*n* = 84).

### 2.3. Historical Control Group

As per the published protocol, this feasibility study included an historical (2015–2017) ‘usual care’ control cohort that was retrospectively identified through the case management database. This cohort included any individual with a primary diagnosis of cancer of working age who had received generic occupational rehabilitation services through IPAR Rehabilitation, the project industry partner.

### 2.4. Beyond Cancer Intervention

#### 2.4.1. Intervention Development

IPAR Rehabilitation, a national provider of OR services within Australia, worked with the research team and a unique stakeholder collaboration to tailor the content of a holistic health coaching program [32] already being successfully used to assist with overcoming work disability to the unique needs of breast cancer survivors. Consumers and cancer support services also provided input into the Beyond Cancer program design, content development and interpretation of feasibility study findings. More details regarding the development of Beyond Cancer and study design are published in our protocol [38].

#### 2.4.2. Beyond Cancer Program—Overview of Content and Delivery Framework

The primary goal of the flexible, tailored participation in the Beyond Cancer intervention was to facilitate work readiness and the gradual return to sustainable, suitable work for each individual. The extent, timing and type of program participation was jointly determined by the OR consultant and the cancer survivor. Intervention components were delivered, as required, in a tailored fashion to breast cancer survivors from around week 5 after referral. Early sessions were used to establish the level of intervention support and services required, as well as the type of services (intervention components) to best support the individual with moving toward work readiness.

**Figure 1:**
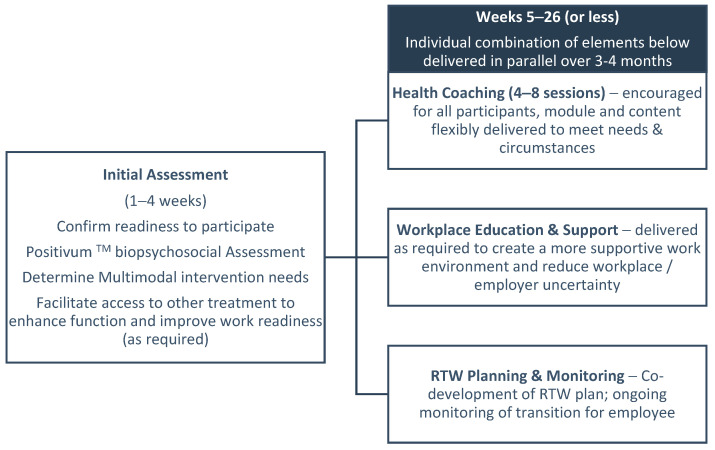
Schematic illustration demonstrating tailored Beyond Cancer intervention delivery.

Due to COVID-19 restrictions and associated vulnerabilities of the cancer population, the Beyond Cancer program was delivered in a tailored fashion using a combination of face-to-face, telehealth and telephone OR consultation sessions by an experienced OR consultant trained in cancer survivorship. The intervention had four main components: The delivery and doses were determined following completion of the psychosocial Positivum^TM^ assessment and an initial discussion between the OR consultant and the survivor. Using the Positivum Assessment, the OR consultant identified key barriers and facilitators to work readiness and tailored the intervention accordingly (see Figure 1).

The Positivum biopsychosocial assessment was used to (1) inform the OR consultants on the survivors’ biopsychosocial needs and (2) as a measure of survivors’ response to the intervention. The Positivum biopsychosocial assessment is a 50-item tool with Likert-style response options developed and tested by our team to determine individuals’ strengths and weaknesses across a range of biopsychosocial constructs. The following psychosocial factors are included: quality of life and general health; pain; physical fatigue; cognitive fatigue; beliefs, perceptions and expectations of health; work and employer; distress and fear of recurrence; empowerment/resilience and perceived support at work. Further information about the validated instruments is provided in our protocol [38]. The assessment provides a high-level individual profile in a standardised report output that is easily interpreted by the trained OR consultant and the cancer survivor, and subsequently used to tailor the intervention services offered during the initial stages of the intervention. The Positivum assessment was delivered online and implemented at referral and again immediately following intervention completion.

### 2.5. Feasibility Analysis

The feasibility analysis aimed to provide indications of intervention effectiveness (through both quantitative work outcomes and qualitative perceptions of effectiveness), and intervention acceptability. Orsmond and Cohn’s [39] feasibility framework was adopted to facilitate this evaluation. This framework identified that the key premise of a feasibility study is to answer the overarching question: Can it work? [39]. Aligning with Orsmond and Cohn [39], the detailed objectives of this feasibility study were:

1. To evaluate the recruitment capability and resultant sample characteristics;
2. To evaluate and refine data collection procedures and outcome measures;
3. To evaluate the acceptability and suitability of the intervention;
4. To evaluate the resources and ability to manage and implement the study and the intervention;
5. To provide a preliminary evaluation of participant responses to the intervention.

The analysis assessed key elements commonly evaluated in feasibility evaluations [39,40]. Table 1 outlines the components of the feasibility analysis, associated questions, as well as data sources and/or collection methods for each component. This section may be divided by subheadings. It should provide a concise and precise description of the experimental results, their interpretation, as well as the experimental conclusions that can be drawn.

### 2.6. Expected Outcomes

The following outcomes were recorded for survivors: return to work outcome, work capacity change and perceived support at work. We also proposed to record the employer’s perspective on acceptability and perceived effectiveness as part of this trial.

#### 2.6.1. Return to Work Outcome

The proposed primary RTW outcome ‘RTW status’ was defined as either ‘no RTW’, ‘RTW with modified hours/duties/role’ or ‘RTW as pre-diagnosis’.

#### 2.6.2. Perceived Support at Work Outcome

The secondary outcome measure ‘perceived support at work’ was included in the protocol to provide an additional quantifiable indication of effectiveness of the Beyond Cancer program. Specifically, the work support factor measured the level of perceived support at the workplace (items are the work-related items from the cancer empowerment questionnaire, CEQ; [41]) at referral and again at intervention completion.

#### 2.6.3. Work Capacity Change

Work capacity change was focused primarily on improving work readiness than a positive return to work per se. Work capacity was considered at referral and post-program completion and categorised as either: (1) no capacity, (2) some capacity but not working (a. not yet ready, or b. ready to consider), (3) certified partial capacity and (4) pre-diagnosis capacity. The determination of capacity was based on both medical certification of capacity at referral as well as a more subjective consideration of capacity from the OR consultant’s perspective that came through in the initial assessment report (an independent rater extracted the work capacity data retrospectively from both groups’ documentation). A positive change or improvement in work capacity was defined for the purpose of this study as a minimum positive shift of 2 steps in classification (e.g., move from no capacity at referral to certified partial capacity or pre-diagnosis capacity). A binomial ‘change in work capacity’ variable classified the change in work capacity from referral to post-program completion as either positive change in work capacity, or no change (or negative change) in capacity.

#### 2.6.4. Acceptability and Perceived Effectiveness from the Employer Perspective

Evidence-based and consumer-informed employer education material was developed for the purpose of this program. As per the protocol we planned to collect data on the acceptability and perceived effectiveness of the intervention from the employer’s perspective [36].

### 2.7. Post-Intervention Survey

All consenting participants were emailed an invitation to complete the evaluation survey containing relevant health education impact questionnaire (heiQ) items (7 of the 9 program evaluation items; [42]). The survey assessed participants’ perceptions and thoughts about the impact of the Beyond Cancer program including their general experience with the intervention, the content, goal-setting, perceived trustworthiness of information and organisation of those delivering the program.

Due to the timing of the program offering (during the COVID-19 pandemic), an open-ended item also assessed whether there were any circumstances that made it difficult for participants to attend their sessions with their consultant.

One final open-ended question asked participants whether they had anything else that they would like to share about their experiences (positive or negative) to help to refine the program.

### 2.8. Interviews and Focus Groups

Interviews and focus groups were conducted with participants and OR consultants, respectively. Interview questions focused on gaining participants’ perspectives on intervention acceptability and suitability and allowed the team to gain a more comprehensive understanding of the perceived effectiveness of Beyond Cancer from a breast cancer survivor perspective. Participants were also invited to comment on how the intervention could be improved.

Focus group questions focused on understanding consultants’ positive perceptions of the benefits of program participation, as well as their perspectives on what needed to be refined in the delivery and implementation of Beyond Cancer.

### 2.9. Data Analysis

Consistent with feasibility study designs, examination of preliminary participant responses to the intervention involved the statistical comparison (chi-square) of changes in high level work status and work capacity outcomes over a similar period of time for the Beyond Cancer participants and a historical usual care cancer survivor group not receiving the multimodal intervention. Repeated measures t-tests also examined whether any psychosocial outcomes showed significant improvement from referral to post-intervention follow up for the intervention group.

For the qualitative data, a systematic, inductive thematic analysis approach aligned with the focus group data analysis framework [43] was used to analyse the data from the consultant focus group and interviews with breast cancer survivors. A reflexive thematic analysis [44,45], appropriate for small and homogeneous samples, was performed, culminating in a final discussion between two independent data analysts and refining of themes until consensus was reached. COREQ guidelines (checklist) [46] were adhered to wherever possible in the context of a feasibility design.

## 3. Results

The results are presented in this manuscript using the Orsmond and Cohn [34] framework for feasibility studies. The following headings are used to be consistent with Table 1 and the structure of the methods: 1A. Recruitment: capability; 1B. Recruitment: sample characteristics; 2. Data collection and outcome measures; 3. Acceptability and suitability; 4. Resources and implementation and 5. Preliminary participant responses.

### 3.1. Recruitment: Capability

A total of 84 females with breast cancer were referred into the program over an extended 24-month period from February 2019 almost exclusively through life insurance. Inadequate referrals could be attributed to the reliance on life insurance as the primary referral pathway, and the overlay of COVID-19-related challenges. The procedural complexities of working within life insurance, as highlighted by the consultant focus group results, as well as the general cautious life insurance climate over this period contributed to a lower rate of referrals than was anticipated. Despite large employers being targeted by the official program launch in May 2019, only three of the eighty-four referrals came directly from the large employer sector. The reliance on life insurance was unintended and is discussed below as a potential major limitation of this study as it limited the heterogeneity of the sample.

### 3.2. Recruitment: Sample Characteristics

The average age of the Beyond Cancer referred cohort was 50.81 years (range 33–67 years, SD = 8.24 years; Table 2). There were two participants over 65 years, and *n* = 10 aged 60 years and above. The historical control group had comparable demographics to the intervention group (Table 2), however, was not matched per se.

Those who did not participate following referral for Beyond Cancer included 15 breast cancer survivors (17.9%) who decided early on not to continue with the program, and a further 14 (16.7%) who reached initial assessment stage before participation was discontinued. For these 29 breast cancer survivors (34.5%), personal circumstances, health status or other perceptions/motivations acted as barriers to proceeding with the Beyond Cancer program. Case management notes and documentation indicated the following reasons for not proceeding:

- Capacity, i.e., major health setbacks and/or treatment barriers (e.g., impending surgery, etc.), not ready for rehabilitation (*n* = 15);
- Secondary and significant mental health barriers (*n* = 3);
- Self-managing well, services not required (*n* = 4);
- Lack of engagement with program, not due to primary condition/treatment (*n* = 7).

Therefore, the primary reasons for individuals not continuing with Beyond Cancer were related to current health and capacity status. That is, the timing during their rehabilitation was such that they were not in a position to benefit greatly, or at all, from the services offered as part of the Beyond Cancer program.

Case notes were reviewed for 84 participants. Challenges were associated with treatment (including post-surgical pain, and chemotherapy/radiotherapy side-effects), fatigue (both physical and mental), anxiety or depression and fear of recurrence. Less commonly noted challenges were related to the survivors’ ‘dependents’ and limited local support due to residential location.

Occupation (Table 3) and rural/remoteness were also considered for the Beyond Cancer referred cohort (using ‘nomenclature of economic activities’, NACE categories, [47]). The two most common occupation sectors were education and health, with many teachers/teacher aides, nurses and allied health workers. The ‘other’ occupation group was also well represented (24.1%) and largely included administrative/clerical employees.

For geographical remoteness [48], a total of 27.0% of survivors referred to the study were classified as ‘inner metro’ residents, 24.3% as ‘outer metro’ and 39.2% as ‘non-metro’. A further 9.5% were ‘unclassified’. Further, 37.8% resided in areas classified as ‘rural or remote’ where residents generally find it harder to access medical assistance and resources.

### 3.3. Data collection and Outcome Measures

In evaluating the appropriateness of the data collection procedures and outcome measures, an initial examination of the completeness of the data set was undertaken. There were two issues identified when considering the appropriateness and viability of the chosen outcome measures:

1. Suitability of the primary RTW outcome measure;
2. Secondary outcome measures: incomplete data set.

#### 3.3.1. Suitability of Primary RTW Outcome Measure

When considering the proposed RTW outcome measures [38], a modified definition of the RTW outcome classification was required. The proposed primary RTW outcome ‘RTW status’ was originally defined as either ‘no RTW’, ‘RTW with modified hours/duties/role’, or ‘RTW as pre-diagnosis’. This was found to be oversimplified and, as a result, the ‘no RTW’ classification (post-intervention) was further sub-divided into those for whom RTW was no longer a viable outcome due to significant ongoing health related concerns directly resulting from the cancer diagnosis or treatment (i.e., RTW not applicable), and those not working at closure with some capacity to return. As discussed at a research team meeting at the commencement of the study, a RTW outcome is only relevant for those whose disease-related health status is compatible with some form of RTW in the near future on which a vocational rehabilitation program such as Beyond Cancer can hope to build. Further, it is impossible to know this at the referral or initial assessment stage as cancer treatment is often continuing and/or follow-up appointments are ongoing to monitor for cancer recurrence.

Therefore, the modified, conditional (excluding those for whom RTW was deemed not applicable) primary outcome of RTW status and a key secondary outcome, RTW capacity, were each contrasted for the Beyond Cancer cohort and the historical control group (see outcome data under Preliminary Participant Responses section below).

#### 3.3.2. Appropriateness of Secondary Outcome Measures

The ‘perceived support at work’ outcome variable.

The secondary outcome measure ‘perceived support at work’ was included in the protocol and recorded. However, the qualitative data demonstrated that breast cancer survivors of this cohort generally reported high levels of workplace/employer support from the outset of their diagnosis and treatment. Therefore, ‘perceived support at work’ might not be an appropriate measure of change for this particular cancer survivor cohort.

*“I just find that it is a different situation having cancer than recovering from an injury from the employer’s perspective..… employers are generally really super supportive of employees with cancer to get back to work.” (Beyond Cancer Consultant);*

*“I didn’t feel like I needed to [engage with the employer support component]. I am sure some people do, but I’m very blessed to be working in the [* *…] sector and they’re very supportive.” (Breast cancer survivor).*

#### 3.3.3. Acceptability and Perceived Effectiveness from the Employer Perspective

In addition, impacted by the unanticipated high levels of perceived support from employers, was the engagement of employers and collection of the employer’s perspective, as per the protocol. This was untenable as the high levels of employer support meant that there was a lack of need for the employer resources and support services. ‘Perceived support at work’ was, therefore, not used as a measure of change for this cohort.

#### 3.3.4. Incomplete Data Set for Secondary Psychosocial Outcome Variables

The remaining Positivum: Cancer Assessment psychosocial factors were also included as secondary outcome measures with potential to provide preliminary indications of the program’s effectiveness. However, only a subset of those who participated in the Beyond Cancer program completed a follow-up Positivum assessment following program completion (*n* = 34 of the 55 participating in the intervention, i.e., *n* = 21 missing data) despite several attempts to follow up with the participants. The implications of this are discussed further below.

### 3.4. Intervention Acceptability and Suitability

Intervention acceptability and suitability was evaluated by examining participant engagement data, and survivors’ and OR consultants’ perceptions of the intervention program.

#### 3.4.1. Participant Engagement

Participant engagement data indicated that a total of 55 of those referred participated in the Beyond Cancer program beyond the initial assessment stage. For these 55 program participants:

- The average program duration was 33 weeks.
- Only 31% (*n* = 17) of program participants completed the program within the proposed upper limit of 26 weeks due to a combination of treatment/health-related intermission periods, and delays associated with COVID-19 restrictions.
- Forty-three of fifty-five (78.2%) showed evidence of participating in at least two of the components of the Beyond Cancer program (e.g., RTW support plus Positivum health coaching); *n* = 15 showed evidence of engaging in two elements, *n* = 19 in three elements, and *n* = 9 in four elements of the intervention.
- The most commonly utilised program element across all 55 program participants was the occupational rehabilitation RTW planning and monitoring services (*n* = 39 of 55, 71%), followed by Positivum: cancer health coaching (35 of 55, 64%) and exercise physiology (*n* = 26 of 55, 47%).
- Only 11 of 55 (20%) chose to engage in the employer education/liaison service provision as a defining feature of the Beyond Cancer program.

#### 3.4.2. Evaluation of the Program

Thirty-six percent of the consenting participants completed the evaluation survey. The survey responses were very positive with mean scores approaching five of a possible six across all seven items, indicative of a high degree of program satisfaction (see Supplement Table S1).

Fifty percent of respondents experienced barriers limiting their attendance, with the most commonly identified barrier being a COVID-19 related issue. Work pressures and commitments and overall health were also identified as potential barriers for a few participants.

In response to the final question on whether they had anything else that they would like to share about their experiences, positive and constructive feedback was received with a few remarking that they were thankful for the opportunity to participate, and one specific comment acknowledging the potential benefits in making this program available more broadly (Table 4).

Seven breast cancer survivors participated in follow-up telephone interviews. All interview participants reported high levels of overall program satisfaction, and that they would highly recommend the Beyond Cancer program to other cancer survivors of working age experiencing a breast cancer diagnosis.

In addition, eight IPAR consultants trained to deliver the Beyond Cancer program, who had experience of delivering the program to breast cancer survivors participated in a focus group. Table 4 summarises feedback received from consultants on the benefits of program participation and outlines areas that could be improved when refining delivery and implementation of the program (Table 4).

Qualitative data collected indicated that the Beyond Cancer program was acceptable and relevant for the breast cancer survivor cohort and consultants delivering the program. The features of the program highlighted as most useful and relevant by consultants and participants included:

- The multimodal nature of the program;
- The focus was holistic and about building work readiness (as opposed to solely RTW);
- The flexible delivery and tailoring to an individual;
- The support, respect and understanding from consultants;
- The utility of the health coaching in identifying key barriers and building work readiness.

In addition, emerging from the qualitative data were a few issues that challenged the intervention delivery in some way; these are summarised under the subsequent feasibility objective.

### 3.5. Intervention Resources and Ability to Manage and Implement the Study and Intervention

In addition to findings that spoke to the acceptability of the program, there were also some learnings emerging from the qualitative data as key themes (Table 4). ‘Challenges to program delivery and implementation’, a theme that emerged from the consultant focus group and participant interviews, is relevant to this part of the feasibility evaluation. These challenges included:

- The difficulty surrounding the timing of referral and when to offer the particular components of the program; this is difficult to get right as the right time varies considerably from one cancer survivor to the next;
- The effectiveness of the consultant training; some found themselves in situations where they felt underprepared, especially regarding emotionally-laden or sensitive topics;
- The potential for additional education and feedback for the life insurance case managers.

Further, the qualitative findings surrounding an additional emerging theme ‘barriers to program participation’ (Table 4) are also relevant in this context. These individual barriers to participation align closely with those that resulted in individuals from discontinuing with the Beyond Cancer intervention (see above), i.e., strong health self-management skills and reduced need for services, and also ongoing issues related to overall health and treatment commitments. Other barriers to participation included current work and other role commitments, and those specific to the COVID-19 pandemic and imposed restrictions.

### 3.6. Preliminary Evaluation of Participant Responses to the Intervention

The preliminary evaluation of participant responses to the intervention was carried out by considering indications of effectiveness (consistent with a feasibility protocol, (34)) by measuring quantifiable changes relative to a historical usual care group in work capacity and primary RTW outcomes (Table 5 and Table 6, and a range of biopsychosocial secondary outcomes from baseline relative to post-intervention follow up for the Beyond Cancer cohort (Figure 1). A binomial ‘change in work capacity’ variable classified the change in work capacity from referral to post-program completion as either a positive change in work capacity, or no change (or negative change) in capacity (Table 5).

Chi-squared analyses demonstrated work capacity improvements for the Beyond Cancer participants that were significantly greater than those seen in the historical usual care group (i.e., 65.5% vs. 32.5%, χ^2^(1, 131) = 14.05, *p* < 0.001).

When statistically contrasting RTW status outcomes, however, the Beyond Cancer cohort showed a non-significant trend toward a higher percentage of positive RTW outcomes compared with those from the historical control group (88.6% vs. 80.82%, χ^2^ (1, 116) = 1.23, *p* = 0.27), as well as a correspondingly lower percentage of those classified as ‘not working’ following the service provision (11.36% vs. 19.18%; Table 6).

Changes in biopsychosocial function.

**Figure 2:**
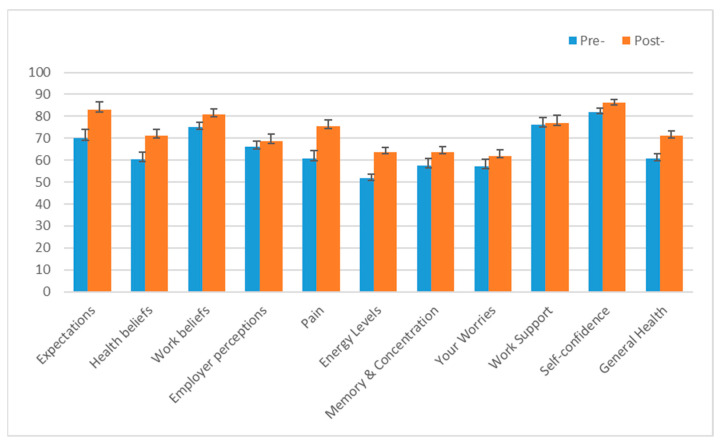
Comparison of average Positivum assessment biopsychosocial factor scores (with SE bars) at referral and follow up for *n* = 34 program participants (*n* = 21 missing follow-up data). Note: the Positivum assessment conducted at follow up was completed anywhere from the initial few weeks following program completion up to 6 months post-program completion.

Another indication of intervention efficacy is the extent to which the Beyond Cancer cohort demonstrated improvements over the course of the program in biopsychosocial function. The results at referral and program completion follow up were compared for *n* = 34 of the *n* = 55 participating cohort (61.8%) of those with Positivum assessment results at referral who had a follow-up Positivum assessment completed following program completion (Figure 2).

Repeated measures *t*-tests were conducted to statistically determine the factors showing a significant improvement. All factors excluding employer perceptions, your worries (fear of cancer recurrence and distress levels) and perceived support at work showed a significant improvement (*p* < 0.05) at follow up. The biopsychosocial factors showing the most substantial significant improvements in order of percentage change (see Figure 2) were:

- Energy levels (physical fatigue symptom severity);
- Pain (current levels of pain and interference);
- Expectations (i.e., confidence that they will be working in the near future);
- Health beliefs (i.e., beliefs about the impact of cancer and treatment on the ability to work);
- General health (perceptions of general health and quality of life).

As anticipated (see above), there was a negligible positive change and a lack of a significant improvement in the work support factor.

Further exploratory analyses (chi-square tests) were conducted to identify whether there was any evidence of significant associations between Positivum assessment factor scores at referral and subsequent RTW outcome and work capacity change. The only factor showing a significant positive relationship found was between RTW outcome and pain, χ^2^(42) = 77.89, *p* < 0.05 (i.e., the higher the pain levels at referral, the less likely a positive RTW outcome).

## 4. Discussion

The overall objective of this study was to determine the feasibility of the Beyond Cancer program to support the transition back to sustainable work and living well for breast cancer survivors. The development of this multimodal intervention to support sustainable return to work for breast cancer survivors was in response to the identification of unmet needs and lack of availability of such services that are multidisciplinary, flexible and tailored to the specific needs of cancer survivors [8,16,30,31]. Critical to the feasibility evaluation is demonstrating preliminary indications of efficacy in the context of relevant work-related and other secondary outcomes for those participating in Beyond Cancer. Participants’ ‘responses to the intervention’ (as per feasibility objective #5) included significantly greater improvements in work capacity as well as greater improvements (though non-significant) in RTW status, relative to the historical usual care group. Further, there were a range of biopsychosocial factors that showed significant improvements from referral to post-intervention follow up for those participating in Beyond Cancer, including improvements in physical fatigue symptom severity, pain (current levels of pain and interference), work expectations, beliefs about the impact of cancer and treatment on the ability to work and perceptions of general health and quality of life.

Study results have been summarised and discussed below as evidence that supports the feasibility of the intervention, i.e., what went well and, subsequently, the challenges and recommendations emerging from the evaluation with reference to the five key feasibility study objectives.

### 4.1. What Went Well

In the context of recruitment capability and implementation, aligning with the first feasibility objective, resources were provided by the industry partner as anticipated and also stepped up as required when referrals were slow and COVID-19 began to impact (i.e., additional resources provided to pursue other potential referral sources). Further, the demographics and sample characteristics (feasibility objective #1) suggest that the intervention participants are reasonably representative of the general population of breast cancer survivors of working age in Australia. Beyond Cancer participants included 65.5% of those referred, with demographics that were comparable with those of an historical control group, i.e., those with primary diagnoses of cancer of working age who had received generic occupational rehabilitation services through IPAR Rehabilitation. Further, the reasons for those not continuing with Beyond Cancer align with those expected to pose significant barriers to participation for breast cancer survivors.

There was also positive indications of acceptability and relevance or suitability for the Beyond Cancer intervention (feasibility objective #3). There was strong participant engagement where (health and treatment-related) circumstances allowed, and survey results showed evidence of high program satisfaction, echoed by the qualitative interview and focus group data. Further, the qualitative data confirmed the value of key characteristics of the Beyond Cancer program that were an integral part of the intervention design. The tailoring and the multimodal nature of the program were demonstrated by the unique ways that participants engaged with the program, with the vast majority of participants taking part in at least two of the multimodal intervention components. Health coaching was the most commonly referenced component throughout the interviews with goal setting repeatedly reported as providing important benefits that enabled heightened motivation around social participation, confidence, adaptation and reassurance. Health coaching was also noted to facilitate the identification of barriers such as cognitive (i.e., concentration and memory) and physical fatigue, loss of confidence and the subsequent normalisation of those symptoms, as well as targeted discussions around ways forward to address or compensate for such challenges.

The vocational support services component was most commonly participated in across the intervention, which aligns with the context of the vocational rehabilitation setting. Importantly though, the trained consultants were able to assist the cancer survivor participants with moving toward a sustainable work goal in such a way that they felt safe and emotionally supported (also addressing feasibility objective #3). Interview data demonstrated that participants felt able to express personal aspects of their lives during the cancer journey and also perceived that their consultants listened and had a good understanding of their needs, challenges and barriers.

### 4.2. Challenges, Learnings and Recommendations

Relevant to the fourth feasibility objective, and as anticipated, program participants experienced a variety of health- and treatment-related barriers to active participation in the intervention at certain stages of their journey as a cancer survivor. These, in combination with the unanticipated COVID-19 related barriers, sometimes affected the participants’ ability to attend sessions in person, and also likely extended the average program duration. Working closely with the life insurance referrer and the cancer survivor participant, these challenges were worked through for the majority of individuals for whom work remained a relevant future goal.

Relevant to feasibility objective #1 and as identified in the limitations, there was a lack of diversity of referral sources and an associated reliance on the life insurance system. This likely introduced a recruitment bias such that this particular life insurance cohort comprised participants with larger and/or government sector employers (health and education sectors) who may have had a better understanding of how to facilitate a return to work following a cancer diagnosis, and who may have also been in a better position to accommodate their return to work. We know from previous research that not all survivors feel that people understand their experiences or receive adequate support to enable RTW [16,28]. Employees of small businesses and those working in less secure employment, e.g., the gig economy, are likely to encounter less supportive attitudes. Indeed, this was found in an earlier pilot of Beyond Cancer (unpublished) which recruited through a different life insurer and found that a lack of employer support for those working in the retail sector was a barrier to participant engagement. This limits the generalisability of these findings to the working breast cancer survivor population as a whole.

A consequence of the identified referral bias was that the majority of individuals felt well supported by their employer. This called into question the relevance of one of the key secondary outcome variables: perceptions of employer support, of relevance to the second feasibility objective (evaluating outcome measures). While the suitability of using this measure as a secondary outcome was deemed not appropriate for this particular cohort of breast cancer survivor participants, the broader literature suggests that it is likely to be relevant and useful for a larger and more representative cohort of cancer survivors who may not be fortunate enough to have such positive experiences post-cancer diagnosis with their employer [49,50,51,52].

In addition, relevant to the second feasibility objective, in particular, challenges surrounding data collection procedures, was the collection of the follow-up Positivum: cancer assessment data which compromised the completeness of the data set (as elaborated below under limitations). As such, appropriate measures are required to ensure that these essential data are able to be captured consistently prior to the next stage national trial. Provision of automatised prompts to complete the final assessment once participants have reached a key milestone (e.g., a specific time period, or a change in work capacity) has since demonstrated some improvement in completion rates.

Relevant to both the first and fourth feasibility objectives (i.e., sample characteristics, and ability to manage and implement the intervention) was the unanticipated challenge of dealing with the implications of the high proportion (38%) of the referred cohort residing in rural or remote areas of Australia. Fortunately, the OR consultants delivering Beyond Cancer were able to pivot to employing a hybrid mode of delivery such that in person or face-to-face sessions were initially prioritised and more flexible modes of delivery, including telehealth and telephone, were utilised to follow up with Beyond Cancer participants as they moved through the program. This worked well for the health coaching component as the content was contained within a take home booklet. The majority of individuals were also able to engage and continue with their exercise physiologist as planned despite this challenge.

### 4.3. Limitations

First, it is important to note that there may be differences between the historical usual care control and intervention groups (e.g., chronology, nature of the disease) despite the demographic similarities.

The sample size for this study was smaller than originally calculated. Participants were recruited through life insurers which may have limited the sample. Additionally, only a subset of those who participated in the Beyond Cancer program completed a follow-up Positivum assessment following program completion despite several attempts to follow up with the participants (*n* = 34 of the 55 participating in the intervention, i.e., *n* = 21 missing data). Lack of follow-up Positivum assessment completion was due to the challenges surrounding post-program assessment compliance of the consultants delivering Beyond Cancer. This represents a common challenge across the occupational rehabilitation sector when attempting to objectively measure effectiveness of OR programs such as Beyond Cancer. Delivering a comprehensive assessment immediately following referral has been established as usual care, which also can be used to inform service delivery and positively influence outcomes. Repeating the assessment upon program completion is, however, not yet accepted as part of usual care despite educating consultants regarding the benefits to the client receiving the services.

### 4.4. Feasibility Study Implications

The Beyond Cancer OR intervention is unique in the way that it brings together the life insurance, occupational rehabilitation and cancer support sectors to benefit breast cancer survivors looking to transition back to sustainable work [38,53]. This study evaluated the feasibility of ‘Beyond Cancer’, a rehabilitation program to support breast cancer survivors to return to health, wellness and work with a representative cohort of breast cancer survivors. Results have demonstrated recruitment and implementation feasibility in the lead up to a full-scale nation-wide trial conditional upon further refining the program based on recommendations emerging from this evaluation.

The most critical limitation to address prior to the launch of the national trial is moving beyond life insurance to increase awareness and gain support from the large employer sector, which also stands to benefit greatly from this program, in order to avoid the referral bias inherent within this study. Partnering with large employers/organisations would importantly enable recruitment outside of the life insurance context. Further, the clear articulation of a viable funding model such that direct referrals can also be received from a variety of contexts, including cancer specialist hospitals, cancer physicians and cancer support organisations, will also help in this regard.

This feasibility study has also contributed to a more in-depth understanding of the individual factors and characteristics that contribute to the successful, staged return to meaningful and sustainable work for breast cancer survivors, thus, adding depth and support for cancer and work models [34,35]. As more research is conducted in this field such as the recent study characterising work ability over time in a young cohort of cancer survivors [20], it is becoming clearly evident that multimodal rehabilitation programs with the flexibility to focus on individualised psychosocial needs are required and should be offered early in the course of the disease.

Further research needs to be conducted to be able to appropriately tailor and adapt vocational rehabilitation programs such as Beyond Cancer to the unique needs of survivors of other cancer types, such as those with head and neck cancers, melanomas and rare cancers [54]. The results of the feasibility study will be disseminated broadly through publication in scientific journals, national and international conference presentations, media releases, national cancer support agencies, stakeholder reports and presentations.

### 4.5. Emerging Recommendations for Beyond Cancer

Finally, a few key learnings emerging from the qualitative data gave rise to the following recommendations that will be implemented to refine and strengthen the Beyond Cancer intervention:

- Moving forward, ensure that adequate care and consideration is given to the timing of referral to services with respect to the cancer survivor ‘journey’ as this can influence engagement; develop ‘case studies’ to demonstrate how timing can positively and negatively influence engagement and use these cases in consultant training;
- Provide an improved resource package to those referring to facilitate a clearer understanding of the aims of the program and better-informed referring;
- Provide more regular communication with referrers to maintain open lines of communication, including more regular updates on cohort progress with attaining program outcomes or goals;
- Further strengthen the consultant training, particularly having more practice in the delivery of unique program components and using scenario-based learning. While there was no negative feedback from those who attended training online (a handful of consultants were from remote locations such as the Northern Territory or Tasmania, and could only attend online), this type of training is likely to be more effective in person;
- Consider additional content to be added to the health coaching as recommended by the cancer survivor participants, e.g., dietary information, content to tackle cancer-related stigmas.

## 5. Conclusions

Feasibility has been demonstrated for this promising multidisciplinary intervention and innovation in breast cancer care focused on returning to good, sustainable work as a critical component of a more holistic rehabilitation for women with breast cancer. While there are several studies that have identified common barriers and unmet needs for cancer survivors when returning to work, and a few proposing what these interventions should include to address unmet needs [14,28,30,31], studies that develop, implement and evaluate such interventions are lacking. This project works toward improved work, health and quality of life outcomes for breast cancer survivors, as well as improvements in practices and service offerings within life insurance, OR and large employer sectors. The next stage will see this intervention further refined to be able to respond to the needs of cancer survivors of various types, and broadly implemented at the national level and with referrals being received from a variety of sources (not wholly reliant on life insurance). Importantly, this multimodal occupational rehabilitation intervention has the potential to improve the work-related and quality of life outcomes of all cancer survivors of working age, their families and society.

## Figures and Tables

**Table 1:** Feasibility analysis framework—components, associated questions and data source/collection methods.

Component	Feasibility Study Question(s)	Data Source/Collection Method
1A. Recruitment: capability	How suitable were the referral sources: consider system structures, funding models and processes?	Numbers recruited vs anticipated; consultant focus group data; research meeting notes
1B. Recruitment: sample characteristics	Are participants appropriate? Do the cohort characteristics suggest it is representative?	Sample characteristics relative to historical control group; review eligibility criteria, reasons for dropping out/discontinuing
2. Data collection and outcome measures	How appropriate are data collection procedures and outcome measures?	Consultant focus group data; completeness of data set; research team meeting notes
3. Acceptability and suitability	Are the study procedures and intervention suitable/acceptable? Are there positive perceptions from the participant perspective?	Participant engagement data; participant interview data and survey responses; consultant focus group data
4. Resources and implementation	Was the intervention implemented as designed? Were resources adequate, including funding, time, training?	Participant interview data and survey responses; consultant focus group data
5. Preliminary participant responses	Are the preliminary outcomes promising or indicative of effectiveness?	Quantitative RTW outcomes and psychosocial assessment outcome data

**Table 2:** Demographics for the Beyond Cancer and historical control group.

	Beyond Cancer Breast Cancer Survivor Cohort	Historical Control Group: Cancer Survivor Cohort
Sample size (n)	*n* = 84 TOTAL *n* = 84 (100%) breast cancer	*n* = 80 TOTAL *n* = 36 (45.0%) breast cancer *n* = 4 (5.0%) colorectal cancer *n* = 6 (7.5%) ‘multilocation’ cancer *n* = 4 (5.0%) leukaemia *n* = 3 (3.75%) lymphoma *n* = 4 (5.0%) prostate cancer *n* = 6 (7.5%) head and neck cancer *n* = 17 (21.25%) other cancers (e.g., kidney, brain, stomach, melanoma)
Mean age (St Dev; Range) No. aged ≥ 60 years No. aged 65+ years	50.81 years (8.24; 33–67 years) *n* = 10 (11.9%) *n* = 2 (2.4%)	50.10 years (9.30; 23–71 years) *n* = 11 (13.75%) *n* = 3 (3.75%)
Program duration, weeks (St Dev; Range)	33.4 (17.91; 11.7–88.3 weeks) *	31.7 (22.97; 9.0–132.4 weeks)

* The program duration (weeks) for the Beyond Cancer cohort was for *n* = 55 participating beyond initial assessment.

**Table 3:** Occupation grouping for the breast cancer survivor referred cohort (*n* = 1 missing data).

Occupation Group	Number of Participants	Percentage
Education	22	26.5%
Health	19	22.9%
Public administration/ defence	5	6.0%
Financial services	5	6.0%
Wholesale/retail/food/accommodation	12	14.5%
Other: Admin/clerical/management/telecommunications	20	24.1%
TOTAL	83	100%

**Table 4:** Themes identified in qualitative data collated from the consultant focus group and breast cancer survivor participant interviews.

Theme	Representative Quote
**Program feedback**	
Perceived benefits of the multi-modal nature (consultant perspective: focus group) Benefits extended beyond RTW goals (participant perspective: interviews)	[the program] *“really was a multi-faceted, sort of, approach and worked really well*”. (Consultant) *“having those face to face discussions were really invaluable…just being able to go meet with someone in person”* (Breast cancer survivor)
Perceived benefits of flexible delivery and tailoring (consultant perspective: focus group)	*“I think the program is so great and ultimately the best rehab is achieved when we can tailor the service to each member …”* (Consultant)
Thankful for support and understanding (survey, participant perspective: interviews)	*“a lovely lady…one of the most beautiful I have met”; “I really did [feel supported]”* (Breast cancer survivor)
Highly recommend Beyond Cancer (participant perspective: interviews)	*“I would fully recommended it to anyone…I didn’t really want to go at beginning. I just thought it would be irrelevant and, you know…just another appointment to go to. But it was so worthwhile,……it really helped me so much*” (Breast cancer survivor)
**Program feedback specific to health coaching**	
Perceived value of health coaching in building work readiness (consultant perspective: focus group)	*“general strategies to assist with the management of fatigue in their everyday lives, not necessarily at work…”* (Consultant) *“the health coaching is what we focused on and she found it useful in terms of building confidence and increasing goal-directed behaviours”* (Consultant)
Other benefits: general emotional support (participant perspective: interviews)	One participant was reassured that their experience was *“quite a normal outcome……feelings were real and this is important [to hear]”.* (Breast cancer survivor)
**Program feedback specific to employer communications**	
Valuable advocate for returning to work (participant perspective: interviews)	*“…appreciated someone speaking ‘on my behalf and tak[ing] any awkwardness out of it’”* (Breast cancer survivor)
Positive engagement with supportive employers (consultant perspective: focus group)	*“...the employers that I dealt with were [sometimes] unsure of the process, but they were amazing…”*⋯(Consultant) *“Her employer was very supportive…and happy to implement any duties or any strategies that the client needed”.* (Consultant)
**Program feedback specific to exercise physiology**	
EPs highly valued (participant perspective: interviews)	*[I got referred to an] “exercise physiologist to try and get back to physical health,...but also managing that fatigue, and it was really good,…enough to help me start to get that strength back without overwhelming me…the focus is now changed…it’s [now] all about increasing bone density.”* (Breast cancer survivor)
**Barriers to participation and engagement**	
Work pressures/commitments (survey; interviews)	*“I had a lot of difficulties because I’m a manager, the expectation is you work full time or not at all”.* (Breast cancer survivor)
Overall health and ongoing treatment (survey; interviews; consultant perspective: focus group)	*“It is just so full on. It is just appointment after appointment. It’s all sorts of things happening and then trying to get through each stage of that initially.”* (Breast cancer survivor)
**Challenges to program delivery and implementation**	
Effectiveness of consultant training—timing/delay; more scenario-based learning (consultant perspective: focus group)	*“I think it’s more just the expectation of what the client’s going through……I found when they start talking about having that constant worry about it coming back, I think we didn’t (or some of us didn’t) have a lot of experience in regards to cancer.”* (Consultant)
Timing of referral and service provision (consultant perspective: focus group; interviews)	*“the program may have been better for her if she was involved right after diagnosis”;* (Consultant) *“I wish I had known about this earlier”;* (Breast Cancer survivor) *“I did have a referral that was sent too early. That individual wasn’t in the space to be able to even think about involvement with us ….”* (Consultant) *“any earlier, I would have still been just coping with [treatment side effects]”;* (Breast cancer survivor)

**Table 5:** Change in work capacity outcomes for the Beyond Cancer and historical control group, including those for whom RTW was ‘not applicable’.

Work Capacity	Beyond Cancer Breast Cancer Survivor Cohort (*n* = 55)	Historical Control Group: Cancer Survivor Cohort (*n* = 77) ^a^
Positive Change capacity	36 (65.5%)	25 (32.5%)
No or Negative Change capacity	19 (34.5%)	52 (67.5%)

**^a^***n* = 3 missing data.

**Table 6:** Comparative RTW status outcomes for the Beyond Cancer and historical control group (excluding those for whom ‘RTW was deemed not applicable’ ^a^.

	Beyond Cancer Breast Cancer Survivor Cohort (*n* = 44)	Historical Control Group: Cancer Survivor Cohort (*n* = 73)
RTW pre-diagnosis hours/duties	18 (45.00%)	34 (46.58%)
RTW partial hours/duties	20 (45.45%)	23 (31.50%)
RTW New employer (Full or partial)	1 (2.27%)	2 (2.74%)
TOTAL positive RTW outcome	39 (88.60%)	59 (80.82%)
Not working at closure/receiving benefits ^b^	5 (11.36%)	14 (19.18%)

^a^ A total of *n* = 11 breast cancer survivors from the Beyond Cancer cohort, and *n* = 7 from the historical control group were excluded from the RTW outcome data as they were classified as ‘RTW not applicable’ at closure. ^b^ These individuals were either not yet working and receiving benefits associated with total incapacity, or job seeking and receiving associated benefits following some program participation.

## Data Availability

The data presented in this study are available on request from the corresponding author.

## Supplementary Materials

The following supporting information can be downloaded at: https://www.mdpi.com/article/10.3390/curroncol30020174/s1, Table S1: Mean scores of the 7 relevant program evaluation heiQ questions.
