# Supplementary material for: ‘Beyond Cancer’ Rehabilitation Program to Support Breast Cancer Survivors to Return to Health, Wellness and Work: Feasibility Study Outcomes
Source: Curr Oncol. 2023 Feb 13;30(2):2249–70. doi: 10.3390/curroncol30020174 (PMC9956005; doi:10.3390/curroncol30020174)
Supplement: Supplementary file 1 [file curroncol-30-00174-s001.zip › curroncol-2119124-supplementary.pdf]

Current Oncology

Supplementals

'Beyond Cancer' Rehabilitation Program to Support Breast Cancer Survivors to Return to Health, Wellness and Work: Feasibility Study Outcomes

Dianne M. Sheppard [1,*], Moira O'Connor [2], Michael Jefford [3,4,5], Georgina Lamb [6], Dorothy Frost [6], Niki Ellis [7] and Georgia K. B. Halkett [8,*]

Supplementary information: HeiQ survey results

All fully consenting participants were emailed with an invitation to complete the evaluation survey containing relevant Health Education Impact questionnaire (heiQ) items. Of those invited, a total of 8 survey completions were obtained (Table S1 below).

The survey, consisting of 7 of the 9 relevant *program evaluation* items of the Health Education Impact Questionnaire (HeiQ, Osborne et al., 2007), assessed participants' perceptions and thoughts about the impact of the Beyond Cancer program including their general experience with the intervention, the content, goal-setting, perceived trustworthiness of information, and organisation of those delivering the program. Each question was responded to using a 6-point Likert scale enabling a basic quantitative analysis of the results.

Table S1. Mean scores of the 7 relevant program evaluation heiQ questions.

Health Education Impact Questionnaire (heiQ) questions	Mean rating (SD)
1. I intend to tell other people that the program is very worthwhile	4.88 (0.6)
2. The program has helped me set goals that are reasonable and within reach	4.38 (1.32)
3. I trust the information and advice I was given in the program	4.88 (0.6)
4. My consultant was very well organised	4.88 (1.17)
5. I feel it was worthwhile my time and effort to take part in the program	4.75 (1.09)
6. Difficult topics and discussions were handled well by my Consultant	4.88 (1.17)
7. I thought the program content was very relevant to my situation	4.50 (1.12)

The above questions were scored using the standard heiQ Likert responses from 1=strongly disagree and 6=strongly agree

The responses for 7 of the 8 survey respondents were very positive with all responding either agree or strongly agree across all 7 items.

In addition to the above items, the survey also asked program participants whether there were any circumstances that made it difficult for them to attend their sessions with their consultant. This standardised drop down response options (of which you could select more than one) included COVID-related issues, transport or parking, remote residential location, or other. Four of the 8 respondents selected COVID-related issues as being a barrier to attendance, and three selected 'other'. The other responses reflected work pressures and commitments (n=2) and overall health (n=1).

Osborne RE, Elsworth GR, Whitfield K. The Health Education Impact Questionnaire (heiQ): An outcomes and evaluation measure for patient education and self-management interventions for people with chronic conditions. Patient Education and Counseling. 2007;66:192-201.
